# 574. Early Post-Marketing Safety Surveillance for the Respiratory Syncytial Virus Prefusion F Protein Vaccine (RSVPreF3 OA)

**DOI:** 10.1093/ofid/ofae631.175

**Published:** 2025-01-29

**Authors:** Céline Verheust, Quentin Deraedt, Philip Bryan

**Affiliations:** GSK, Wavre, Belgium, Wavre, Vlaams-Brabant, Belgium; GSK, Wavre, Belgium, Wavre, Vlaams-Brabant, Belgium; GSK, London, United Kingdom, London, England, United Kingdom

## Abstract

**Background:**

RSVPreF3 OA (GSK) received its first approval on 3 May 2023 in the United States (US), for the prevention of lower respiratory tract disease caused by respiratory syncytial virus (RSV) in ≥ 60-year-olds. By 15 March 2024 (data lock point [DLP] of this analysis), RSVPreF3 OA was also approved in all European Economic Area countries, and > 10 additional countries across both hemispheres. We summarized the post-marketing safety surveillance data following vaccination with RSVPreF3 OA.

**Methods:**

Exposure was based on RSVPreF3 OA doses distributed and administered. The spontaneous adverse events (AEs) reported from 3 May 2023 up to DLP were retrieved from GSK’s Global Safety Database. Observed-to-expected (O/E) analyses were performed for reports of Guillain-Barré syndrome (GBS) and transverse myelitis.

**Results:**

Up to DLP, > 9.1 million RSVPreF3 OA doses were distributed globally, of which > 7.8 million doses were administered to adults in the US. There were 1,301 reports including 3,278 AEs following RSVPreF3 OA administration, of which 91.34% were considered non-serious (Table). Most reported AEs were consistent with the expected reactogenicity profile, as observed in clinical trials and listed in the prescribing information. O/E analysis of GBS (N=9) and transverse myelitis (N=1) found that the reporting rates did not exceed the expected background incidence (16 and 6 expected cases, respectively); no other neuroinflammatory events were reported. Nineteen reports of atrial fibrillation were reported and more plausibly reflect the epidemiology of the older adult population being vaccinated. Of all reports, 539 (41.43%) described medication errors, of which 85.34% were without associated symptoms. Although RSVPreF3 OA is not indicated for pregnant women, 263 cases of exposure during pregnancy (48.79% of the medication errors) were reported.

**Conclusion:**

Based on the data from the first 10 months of post-marketing surveillance, the safety profile of RSVPreF3 OA was shown to be consistent with that observed in clinical trials. The available evidence does not suggest a causal association between RSVPreF3 OA and neuroinflammatory events or atrial fibrillation. Along with the vaccine efficacy shown in clinical trials, the data supports a favorable benefit-risk profile.

Funding: GSK

**Disclosures:**

**Céline Verheust, PhD**, GSK: Salary as GSK employee, with stock options included|GSK: Stocks/Bonds (Public Company) **Quentin Deraedt, PhD**, GSK: Salary as GSK employee, with stock options included|GSK: Stocks/Bonds (Public Company) **Philip Bryan, PhD**, GSK: Salary as GSK employee, with stock options included in salary|GSK: Stocks/Bonds (Public Company)

